# Alterations in Lipid Levels of Mitochondrial Membranes Induced by Amyloid-**β**: A Protective Role of Melatonin

**DOI:** 10.1155/2012/459806

**Published:** 2012-05-16

**Authors:** Sergio A. Rosales-Corral, Gabriela Lopez-Armas, Jose Cruz-Ramos, Valery G. Melnikov, Dun-Xian Tan, Lucien C. Manchester, Ruben Munoz, Russel J. Reiter

**Affiliations:** ^1^Department of Cellular and Structural Biology, University of Texas Health Science Center at San Antonio, 7703 Floyd Curl Dr., San Antonio, TX 78229, USA; ^2^División Neurociencias, Centro de investigación Biomédica de Occidente, Instituto Mexicano del Seguro Social, Sierra Mojada 800 Col. Independencia, 44340 Guadalajara, JAL, Mexico; ^3^University Center for Biomedical Research Center, University of Colima, Colima, COL, Mexico; ^4^Departamento de Química, Centro de Ciencias Exactas e Ingenieria (CUCEI), Universidad de Guadalajara, Blvd. Marcelino García Barragan 1421, 44430 Guadalajara, JAL, Mexico

## Abstract

Alzheimer pathogenesis involves mitochondrial dysfunction, which is closely related to amyloid-**β** (A**β**) generation, abnormal tau phosphorylation, oxidative stress, and apoptosis. Alterations in membranal components, including cholesterol and fatty acids, their characteristics, disposition, and distribution along the membranes, have been studied as evidence of cell membrane alterations in AD brain. The majority of these studies have been focused on the cytoplasmic membrane; meanwhile the mitochondrial membranes have been less explored. In this work, we studied lipids and mitochondrial membranes *in vivo*, following intracerebral injection of fibrillar amyloid-**β** (A**β**). The purpose was to determine how A**β** may be responsible for beginning of a vicious cycle where oxidative stress and alterations in cholesterol, lipids and fatty acids, feed back on each other to cause mitochondrial dysfunction. We observed changes in mitochondrial membrane lipids, and fatty acids, following intracerebral injection of fibrillar A**β** in aged Wistar rats. Melatonin, a well-known antioxidant and neuroimmunomodulator indoleamine, reversed some of these alterations and protected mitochondrial membranes from obvious damage. Additionally, melatonin increased the levels of linolenic and n-3 eicosapentaenoic acid, in the same site where amyloid **β** was injected, favoring an endogenous anti-inflammatory pathway.

## 1. Introduction

 We hypothesized that due to its amphipathic nature [1], its physicochemical functions [2] and aided by the induced oxidative stress [3, 4], A*β* paves its own pathway from the extracellular space to the mitochondria where it disrupts membrane fluidity and causes energetic dysfunction. This mechanism of membrane permeabilization induced by A*β* and its own internalization might be the major cause of mitochondrial dysfunction. 

Following injection of A*β*_1–42_ into the hippocampus of healthy Wistar 6-month-old rats, we reported deposits of this peptide fragment forming plaques in the extracellular space. Thereafter A*β* was observed in cytoplasmic membranes, especially those of axons, where it accumulated in one or two poles of the axons giving the appearance of onion bulbs, as observed in demyelinating pathologies. Demyelination is also a feature of Alzheimer's disease (AD) [5, 6]. It is noteworthy that the animals used in the experiments had no other condition or genetic predisposition to form plaques or other AD features. A*β* peptide then appeared in the cytoplasm, and finally in mitochondrial membranes, where its presence was associated with mitochondrial dysfunction. The question remaining is what alterations A*β* produce in the lipid composition of mitochondrial membranes, particularly in those fatty acids and phospholipids previously related to the A*β* pathogeny. Moreover, if A*β*-induced oxidative stress plays a key role in damaging lipids in cell membranes, what is the potential role of the antioxidant melatonin when this process affects mitochondrial membranes?

 Lipid and fatty acid changes have been studied primarily in plasma membranes or in synaptosomes prepared from AD postmortem brains; those changes have been associated with aging, A*β* deposits, dementia, and even with mild cognitive impairment. Other results have been obtained using purified synaptosomal plasma membranes from transgenic mouse brain. To our knowledge, no studies have focused on mitochondrial membranes in response to *in vivo* extracellular deposits of fibrillar A*β*. This approach has relevance to the aforementioned hypothesis and our previous results.

The lipid sensing and lipid-regulated proteolysis is a well-characterized phenomenon. This involves the processing of the sterol regulatory element binding proteins (SREBPs), as the best known example [7]. Likewise, saturated fatty acids are related both to amyloidogenesis and to tau hyperphosphorylation. Palmitic acid (PA) is related to the *β*-site amyloid precursor protein (APP) cleaving enzyme (BACE1) upregulation and amyloidogenic processing of APP in primary rat cortical neurons [8]. Also, cortical neurons growing in conditioned media from astrocytes treated with palmitic acid and the unsaturated oleic acid expressed hyperphosphorylation of the tau protein. This was later found to be an oxidative-related phenomenon since the addition of the antioxidant N-acetyl cysteine reduced hyperphosphorylation of tau [9]. Changes or reaccommodations of fatty acids within the lipidic structures are continuous, and this may represent oxidative stress-induced remodeling [10]. However, an AD-specific or an A*β*-induced specific change does not exist. Nonetheless, it is also known that unsaturated fatty acids, specifically oleic and linoleic acids, may stimulate presenilin-1 levels and *γ*-secretase activity, which participate in the generation of A*β* [11].

The relationship between oxidative stress, peroxidation of membrane lipids, and neurodegeneration has been primarily explored in postmortem studies, where a significant decline in polyunsaturated fatty acids (PUFAs), especially arachidonic and docosahexenoic acids, has been found [12]. These changes are directly related to the augmentation in 4-hydroxynonenal, a toxic by-product of the peroxidation of membrane PUFAs, especially arachidonic acid (AA) [13]. In fact, A*β* may induce the phospholipase A2, responsible for AA release from cytoplasmic membranes [14]. The observed drop in docosahexaenoic acid (DHA), on the other hand, is correlated with the dietary intake of DHA which may reduce the amyloid burden [15] by stimulating the nonamyloidogenic processing of APP [16]. Fatty acids modulate the production and activity of a variety of neurotransmitters and the alterations of fatty acids in the diet of rodents have been demonstrated to result in changes in the ability of the animals to learn or retain new information [17, 18]. The ratio between unsaturated and saturated FA (U/S) expresses the degree of unsaturation being linked to less membrane fluidity as an adaptative phenomenon. Changes in the ratio of these fatty acids have been thought to be involved in a variety of diseases including cancer, diabetes, and neurologic diseases. The overproduction of A*β* leads to a decline in Δ-9 desaturase activity with an alteration in membrane fatty acids This results in altered membrane mobility leading to a decline in neurotransmitter activity and a decreased release of acetylcholine [19].

 It has been demonstrated that A*β* peptides interact with anionic lipids which leads to a significant alteration in the properties of the bilayer itself. Phosphate groups in anionic lipids and aliphatic aminoacids (Val-Val-Ile-Ala) at the C-terminal end of A*β* mediate that interaction while oxidative stress induces a significant rise in anionic phosphatidylserine (Ptd-Ser). Membrane disruption induced by A*β*-peptide is mediated through perturbations of the lipid order caused by interaction of peptides with head groups and/or formation of micelles [20]. Reciprocally, when incubated with Ptd-Ser, A*β* undergoes transformation from a random coil to a *β*-structure [21]. Furthermore, there seems to be a cell selective neurotoxicity due to A*β* determined by surface Ptd-Ser, apart from the levels of ATP [22]; this also relates to our hypothesis. Thus, the capacity of cells to bind A*β* seems to be associated with cells with expressed measurable Ptd-Ser on the membrane surface, and this feature is correlated with apoptotic signaling. It should be noted that Ptd-Ser is normally found on the inner face of the surface membrane of healthy cells. In the early stage of apoptosis, however, or under specific stimulation, such as Ca^2+^influx, a hallmark in the mitochondrial pathogenic mechanisms possibly involved in AD [23, 24], Ptd-Ser can translocate to the outer leaflets of the plasma membrane and be exposed to the extracellular space. Thus, Ptd-Ser becomes a surface receptor site for A*β* binding, in such a manner that annexin or apolipoprotein E2, proteins with the ability to interact with Ptd-Ser, may protect neurons from A*β* neurotoxicity [25]. Since Ptd-Ser is an anionic phospholipid, it may produce an acidic local environment, which is optimal for aggregation of the A*β* peptide [26].

 Phosphatidylcholine (PtdChol) is a major constituent of cell membranes, commonly found in the outer leaflet, and it is a particular target for A*β*. For example, evaluated in zwitterionic bilayers based on PtdChol membranes, A*β* associates with lipid heads, and when fused into a zwitterionic planar bilayer, it is rapidly transformed from helical- to *β*-structure and exhibits a channel-like behavior [27]. In this manner, A*β* disturbs intracellular calcium homeostasis because it renders lipid bilayers permeable to ions.

Changes in packing and orientation of phospholipids in membranes is a phenomenon promoted by cholesterol, which modulates the membrane binding of amyloidogenic proteins [28, 29]. It has been documented that cholesterol increases the binding of A*β* to lipid membranes [30, 31]. The concentration of cholesterol in the membranes has been related to the extent and depth of insertion of A*β* into the membrane [32]. However, a computational study using PtdChol and PtdChol/cholesterol bilayers, which mimic the cholesterol depleted and enriched lipid domains of neuronal membranes, revealed a protective role of cholesterol in preventing both A*β*-induced membrane disruption and membrane surface-induced *β*-sheet formation [33]. Nonetheless, by electron paramagnetic resonance spectroscopy, it has been demonstrated how, driven by hydrophobic interactions, A*β* is inserted into bilayers, between the outer part of the hydrophobic core and the external hydrophilic layer. This causes displacement of cholesterol towards the more external part of the membrane where the crowding of cholesterol in turn causes membrane rigidity in this region of the bilayer [34]. This membrane rigidity has been demonstrated in mitochondria obtained from postmortem AD brain [35]; importantly, alterations in mitochondrial membrane fluidity are primarily related to lipid peroxidation [36], which, again, emphasizes the importance of oxidative stress.

Since oxidative stress is a major event in AD progression and is especially related to membrane dysfunction, mitochondrial failure, and apoptosis, the antioxidant melatonin has proven useful in delaying the progression of damage in AD [24]. We have demonstrated in *in vivo* experiments after the injection of A*β* directly into the hippocampus that orally administered melatonin is more effective in reducing oxidative stress than are vitamins C and E [4]. The effect of melatonin has been shown to be especially protective for PUFAs during nonenzymatic lipid peroxidation [37], as observed in transgenic mouse model of AD [38]. Melatonin also may preserve arachidonic and docosapentaenoic acids as observed during ascorbate-Fe^++^ peroxidation in rat testicular microsomes and mitochondria [39].

The current work is based in those fatty acids or lipids previously reported to be involved in A*β*-lipid interactions and the protective effect of melatonin on A*β*-induced membrane disruption; this latter process is mediated through perturbations of the lipid order caused by an interaction of peptides with head groups and/or formation of micelles [20]. Our results correspond exclusively at the region of the hippocampus where the A*β* was injected.

## 2. Materials and Methods

### 2.1. Animals and Experimental Design

Surgical and animal care procedures were performed with strict adherence to the guide for the Care and Use of Laboratory Animals (National Institutes of Health, publication number 86–23, Bethesda, MD, USA). All protocols and procedures were approved by the institution's Animal Care and Use Committee. Male Wistar rats (250–280 grams; 3-month-old) were housed in pairs in a colony room on a 12:12 dark/light cycle with lights off at 20:00 h; food and water were provided ad libitum. The rats were divided (*n* = 5) into the following groups: (1) PBS-injected rats, (2) fibrillar A*β*_1–42_-injected rats (fA*β*), and (3) H_2_O_2_ (200 *μ*M) intracerebrally injected rats. Two additional groups, fA*β*+Mel and H_2_O_2_+Mel, were included. In this case, the fA*β* or H_2_O_2_-intracerebrally injected animals received antioxidant treatment with melatonin (Sigma, St. Louis, MO, USA) dissolved in the drinking water to yield an estimated daily dose of 20 mg/kg/day. IPBS was used as control instead of A*β* peptides since even nontoxic A*β* derivatives, such as the scrambled A*β* usually employed as control in *in vivo* models, may themselves produce free radicals [40, 41]. H_2_O_2_ was chosen as a positive control because of its close relationship with A*β* pathogeny [42]. H_2_O_2_ is considered its principal mediator [43] and secondary messenger of death signals [44]. Additionally, H_2_O_2_ accumulates in mitochondria long before the appearance of A*β* plaques in the extracellular space as evaluated in Tg2576 mice [45].

### 2.2. Brain Coordinates for Hippocampal Injections

Hippocampal injections of A*β*_1–42_ (2 *μ*L at a final concentration of 1 mM) were performed as previously described [4, 46, 47]. Lyophilized synthetic A*β*_1–42_ (Sigma, St. Louis, MO, USA) peptide was solubilized (10^−4 ^M) in filtered PBS; it was then allowed to incubate with continuous agitation (Teflon stir bar at 800 rpm) at 23°C for 36 h in order to form fibrillar aggregates. Rats, anaesthetized with chloral hydrate (350 mg/kg, i.p.), were placed in a stereotaxic instrument for intracerebral injection over a 5 min period (coordinates: anterior-posterior = −3.8 mm, medial-lateral = 2.0 mm, dorsal-ventral = 2.6 mm from bregma; this corresponds to the CA1 region as determined by the atlas of Paxinos and Watson [48] as a guide) using 5 *μ*L Hamilton microsyringe coupled to a 30-gauge needle through flexible tubing. The needle was left in place for 5 min after the injection. The same coordinates were used for experiments with H_2_O_2_. 36 hours after the injections, rats were deeply anesthetized and transcardially perfused with 200 mL of PBS. Those animals used for immunohistochemical procedures were additionally perfused with 4% paraformaldehyde. The rats were sacrificed by decapitation and the brain was removed immediately, placed in cold PBS, and a piece of tissue (164–180 mg), including the lesioned area, was taken with a punch (diameter 10 mm), at the base of the needle tract. This piece included the hippocampus and adjacent cortical areas.

### 2.3. Immunoelectron Microscopy

For immunoelectron microscopy, the hippocampal tissue samples were fixed in 4% paraformaldehyde for 24 hours and immersed in 2.3 M sucrose solution for 24 hours. Thereafter, small blocks were cut and postfixed in osmium tetroxide (2% in PB 0.2 M) for 45 minutes and then embedded for 48 hours in Embed 812 (Electron microscopy Sciences). Ultrathin sections of 70–90 nm were cut with an ultramicrotome (Reichert Om3) and mounted on nickel grids and then incubated for 2 hours in 5% BSA and 0.1% fish gelatin. For the immunolabeling experiment, the mounted sections were then incubated for 24 hours at 4°C with the primary polyclonal antibody Anti-*β*A (Anti-*β*A42, from Santa Cruz) at a dilution 1 : 1000, then washed four times with PBS 0.1 M and 0.1% tween-20, and further incubated for 3 hours at room temperature with a 6 nM gold-conjugated secondary goat anti-rabbit antibody (Jackson Immunoresearch Laboratories) at a dilution of 1 : 500. After four washes with PBS, sections were counterstained with uranyl acetate (2%) for 15 minutes and lead citrate for 5 minutes and examined in a Zeiss EM 906 transmission electron microscope (Oberkochen, Germany).

### 2.4. In Vivo Analysis of Mitochondrial Free Radicals

Analysis of mitochondrial free radical generation-Mitotracker red CM-H2XRos (Molecular Probes), a rosamine derivative used to detect mitochondrial free radicals, was diluted in DMSO to form a 1 mM stock solution. 100 *μ*L of that solution was diluted in 5 mL of physiological saline and stored sterile at 4°C as a working solution. Applied at a dose of 0.030 *μ*g/kg, CM-H2XRos did not affect the functional properties of mitochondria after loading, since neither the respiratory output nor cell viability was significantly changed, as evaluated in a separate study (data not shown). Two hours following the intraperitoneal injection of CM-H2XRos, animals were perfused transcardially with PBS followed by 4% paraformaldehyde. The brain was immediately removed and immersed in the fixative for 8–10 h. Following a brief washing in PBS, brain slices were cut into 25–30 *μ*m thick sections, including the area of interest, with the vibratome and incubated free-floating in Mito Tracker Green (Molecular Probes, Ex/Em 490/516 nm), which selectively stains mitochondria both in live cells and in cells that have been fixed. Then sections were mounted on adhesive (Vecta Bond) coated glass slides, with a DNA dye, 4′,6 diamidino-2-phenylindole (DAPI), containing mounting medium (Vectashield, Vector Laboratories) in order to evaluate mitochondrial mass in cells with nuclear counterstaining in blue (Ex/Em 359/461 nm). The mitochondrial free radicals were analyzed by monitoring the oxidized fluorescence product (Ex/Em 554/576 nm) of CM-H2XRos under a fluorescence microscope. Integrated optical density (IOD), number of mitochondria, and its mitochondrial area were determined by using image analysis software (Image-Pro Plus v5.0). Results are presented here as a CMH2XRos/MitGreen IODs quotient.

### 2.5. Mitochondrial Isolation

For mitochondrial isolation, briefly, brain tissue was minced and placed in prechilled Dounce homogenizer with SHE buffer (0.25 M sucrose, 5 mM HEPES and 1 mM EGTA,. PH 7.4), followed by centrifugation at 2,500 rpm for 10 min, 4°C, and recentrifugation of the supernatant (8,500 rpm, 10 min), to obtain a crude mitochondrial pellet. Following a 10 min incubation in ice, the pellet was resuspended again in SHE plus delipidizedbovine serum albumin (Sigma Chemical Company). Albumin was eliminated by centrifugating this suspension of mitochondria at 9,500 rpm for 10 min. The protein content in the mitochondrial fraction was determined by Lowry's method [49].

### 2.6. Fluidity Changes of Mitochondrial Membranes

1,3 dipyrenylpropane (DPP) incorporation into membranes to form intramolecular excimers depends mainly on medium microviscosity and temperature of determination (24). Membrane fluidity is determined by estimating the excimer to monomer fluorescence intensity ratio (Ie/Im) of this fluorescent probe, a quotient that reflects lateral mobility of membrane phospholipids (25). Briefly, mitochondria were resuspended in Tris-HCl buffer (50 mM, pH 8) and then fragmented by sonication for 15 seconds before being separated by centrifugation at 13,000 rpm. The mitochondrial membrane pellet was resuspended and proteins were measured by Lowry's method. 0.1 mg of mitochondrial protein was mixed in a spectrofluorometric cell containing Tris-HCl (20 mM, pH 7.5). DPP solution in ethanol of spectroscopic grade was diluted (0.02 mg/mL) and mixed with membranes given a molar ratio of fluorescent probe to membrane phospholipids of 1 : 1400; these mixtures were incubated in darkness for 4 hours at room temperature. Fluorescence of DPP incorporated into membranes was measured at 24°C on a Perkin Elmer fluorescence spectrometer, LS50B. The fluorophore was excited at 329 nm and the monomer and excimer fluorescence intensities were read at 379 and 480 nm, respectively.

### 2.7. Chromatographic Analysis of Fatty Acids

Fatty acids from membranes were extracted with chloroform:methanol (2 : 1 vol/vol) and analyzed by gas-liquid chromatography. Briefly, C17:0 heptadecanoic acid, as internal standard, was added to 1 mg of mitochondrial protein and the mixture of methanol and chloroform, both dissolved in BHT, was added. After centrifugation, the chloroform phase was extracted and a second extraction was done by adding anhydrous sodium sulphate. The extract was evaporated under nitrogen, reacted with a mix of methanol, sulfuric acid, and toluene at 90°C for 2 hours, and then redissolved in hexane and a 5% saline solution. Following the extraction of the organic phase, the hexane was evaporated under nitrogen to obtain derivatized fatty acids to be placed into the injector of a Carlo Erba gas chromatograph with flame ionization detection; the temperature of the injector was 250°C and the oven temperature was maintained at 196°C, using helium as a carrier gas at 1.4 kg/cm^2^.

### 2.8. Chromatographic Anaylsis of Phospholipids

Phospholipids were extracted with a methanol/chloroform solution mixed in a 2 : 1 ratio, dried in a SpeedVac, and then redissolved in chloroform. Following a second extraction adding anhydrous sodium sulphate, the solution was filtrated and then evaporated. Samples were analyzed by high-pressure liquid chromatography. Results are provided in relative percentage from the correspondent areas in the chromatogram.

### 2.9. Chromatographic Anaylsis of Cholesterol

Membrane lipids were extracted with chloroform:methanol (2 : 1 vol/vol) and analyzed by gas-liquid chromatography. Briefly, 10 *μ*g of stigmasterol, as internal standard, was added to 1 mg of mitochondrial protein and the mixture of methanol and chloroform, both dissolved in BHT, was added. Extracted lipids react for 1 hour at 60°C with a mix of hexamethyldisilazane, trimethyl fluorosilane, and dry pyridine to convert free cholesterol and stigmasterol in their corresponding trimethyl esters. The mixture was evaporated with nitrogen and then redissolved in hexane to be injected into the Carlo Erba gas chromatograph with flame ionization detection; the temperature of the injector was set to 275°C, the temperature of the detector was 260°C, and that of the oven was maintained at 275°C. Helium was used as a carrier gas at 1.5 kg/cm^2^.

### 2.10. Statistical Analysis

All data are shown as means ± SE of triplicate experiments. Statistical analysis of the data for multiple comparisons was performed by two-way ANOVA followed by Student's tests. For a single comparison, the significance of any differences between means was determined by unpaired *t*-tests. The criterion for significance was *P* < 0.05 in all statistical evaluations.

## 3. Results

### 3.1. A*β* at the Brain Enters the Neurons and Eventually Presents in Mitochondrial Membranes

**Figure 1:**
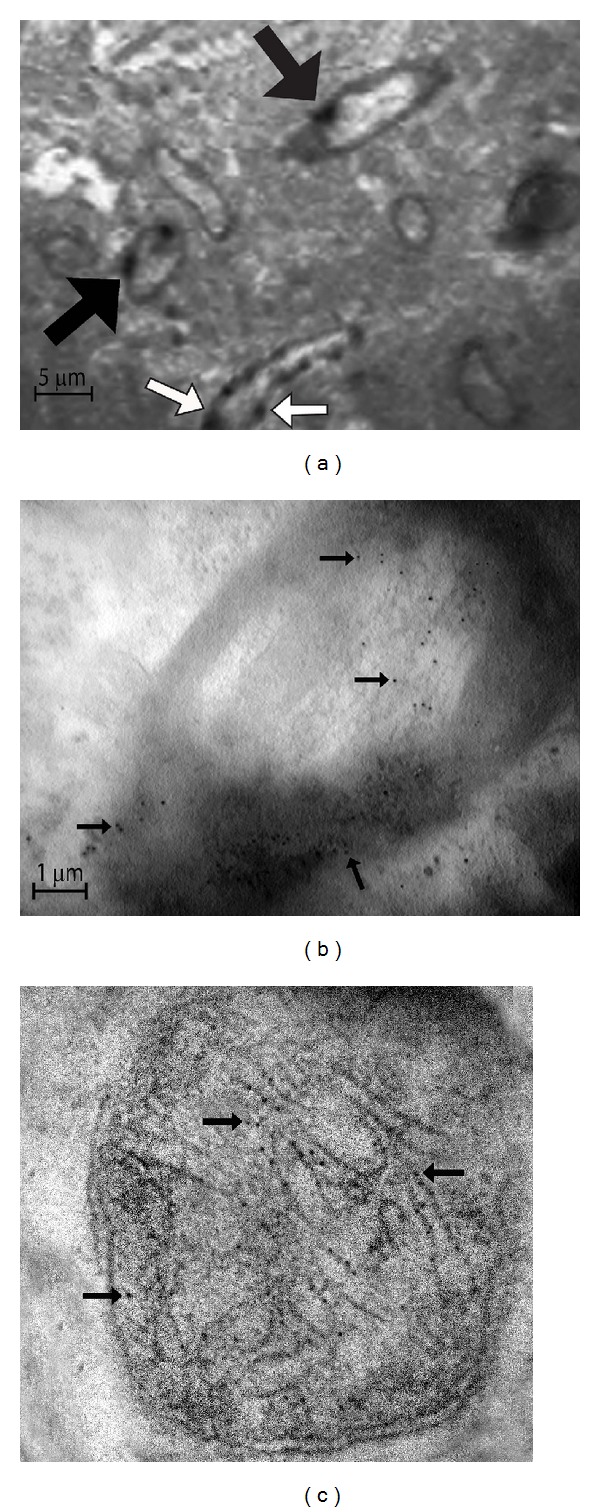
A*β* stain by immunoelectron microscopy. 36 hours after the intracerebral injection of A*β* tissues from the injected area were obtained and subjected to immunohistochemistry by using a primary polyclonal antibody against A*β*. Deposits of A*β* forming deposits in the extracellular space were revealed by conventional light microscopy (data not shown). A*β* immunoreactivity was then revealed with a 6 nm gold label and observed in a transmission electron microscope which allows us to identify (a) deposits of A*β* within myelin axons (black arrows) and in the vasculature (white arrows). (b) Deposits of A*β* (black arrows) penetrate the axon membranes causing demyelination and appear in the axons. Axons look like bulb onions. (c) A*β* appears within the mitochondria finally, where it forms deposits along the cristae (black arrows) and causes intense inflammation, destruction of membranes, and vacuolization (magnification at 27800x).

12, 24, 36 and 48 hours following the intracerebral injection of fibrillar A*β*, deposits of A*β* forming aggregates were reactive to A*β* polyclonal antibody and revealed by immunohistochemistry. Congophilic amyloid deposits remained visible up to 21 days following the intracerebral injection (data not shown). Tissue sections of 50 *μ*m, obtained with a vibratome, were used for immunoelectron microscopy and the A*β* positive immunoreactions were observed in mitochondria along the membranes and deep in the cristae. The presence of A*β* deposits in mitochondria was accompanied by a significant lost of their architecture, characterized by swelling, broken cristae, lost of membrane integrity, and vacuole formation (Figure 1).

### 3.2. The Lost of the Cytoarchitecture Was Related to Free Radical Overproduction

**Figure 2:**
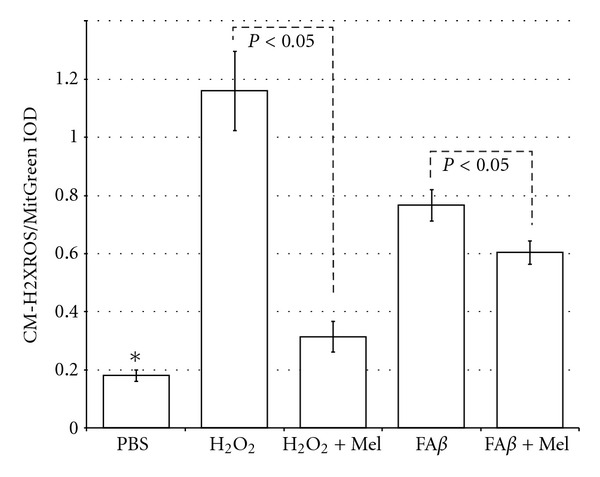
Compared with PBS-injected brains, those brains injected with A*β* or with H_2_O_2_ had a significant increase in free radical levels in mitochondria, according to the CM-H2XROS/MitGreen quotient (*P < 0.05 versus all the other groups). However, by using melatonin a significant decrease in mitochondrial free radicals was observed both in A*β*- and in H_2_O_2_-injected brains.

CM-H2XROS is a reduced, non fluorescent X-rosamine derivative, which is sequestered by mitochondria where it is retained and oxidized. Under oxidation, CM-H2XROS emits fluorescence as a consequence of the number of free radicals produced by mitochondria. This reagent is normally used in *in vitro* experiments after adding it to cells in culture. To demonstrate the effects of A*β* *in vivo,* we have introduced a variant by injecting CM-H2XROS intraperitoneally 15 minutes before tissue collections (as explained). Once the tissue was obtained, sections of the lesioned area were immediately cut in a vibratome and stained with Mito Tracker Green (MitGreen), which is essentially nonfluorescent in aqueous solutions, only becoming fluorescent once it accumulates within the lipid environment of the mitochondrion. Thus, the CM-H2XROS/MitGreen IOD quotient identifies the quantity of free radicals by the mitochondria present on each field of the microscope. We found a significant overproduction of free radicals both in A*β*- and in H_2_O_2_-treated brains (*P* < 0.05) as compared to PBS-injected brains. Brains of animals who had received melatonin showed significantly lower levels of free radicals (*P* < 0.05) (Figure 2).

### 3.3. Membrane Fluidity Was Inversely Correlated to Free Radical Overproduction

**Figure 3:**
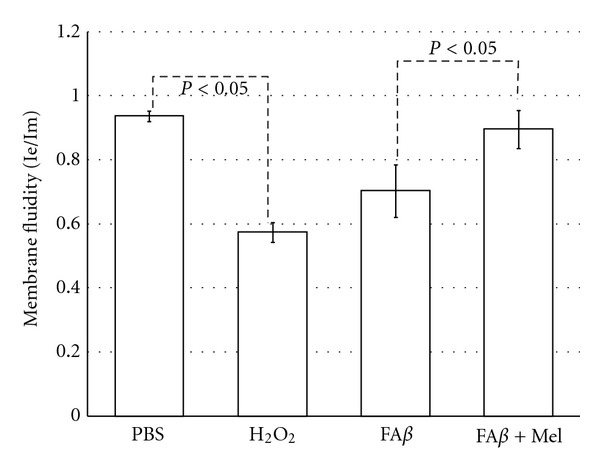
Brains of animals injected with A*β* showed a significant reduction in membrane fluidity as compared with PBS-injected brains, although less obvious than the observed in H_2_O_2_-injected brains, used as a positive control, which is in concordance with the degree of the free radicals overproduction, as shown in the previous graphic. Membrane fluidity in animals receiving melatonin was restored at the same level than the PBS group.

The highest value in membrane fluidity was observed in PBS-injected brains, which showed the lowest amount of free radicals as well (Figure 3). The highest overproduction of free radicals, according to the CM-H2XROS/MitGreen IOD quotient, was observed in H_2_O_2_-injected brains. Interestingly, even though the production of free radicals in brains injected with fA*β* was significantly higher than the quantity of free radicals observed in the PBS group, the difference between membrane fluidity and free radical overproduction was less obvious, as compared with the positive control group of H_2_O_2_. Brains of animals treated with melatonin had significantly reduced levels of free radicals and the difference between these and membrane fluidity was again obvious (Figure 3).

### 3.4. The Unsaturated/Saturated Ratio, Significantly Affected by A*β*, Is Restored by Melatonin

**Figure 4:**
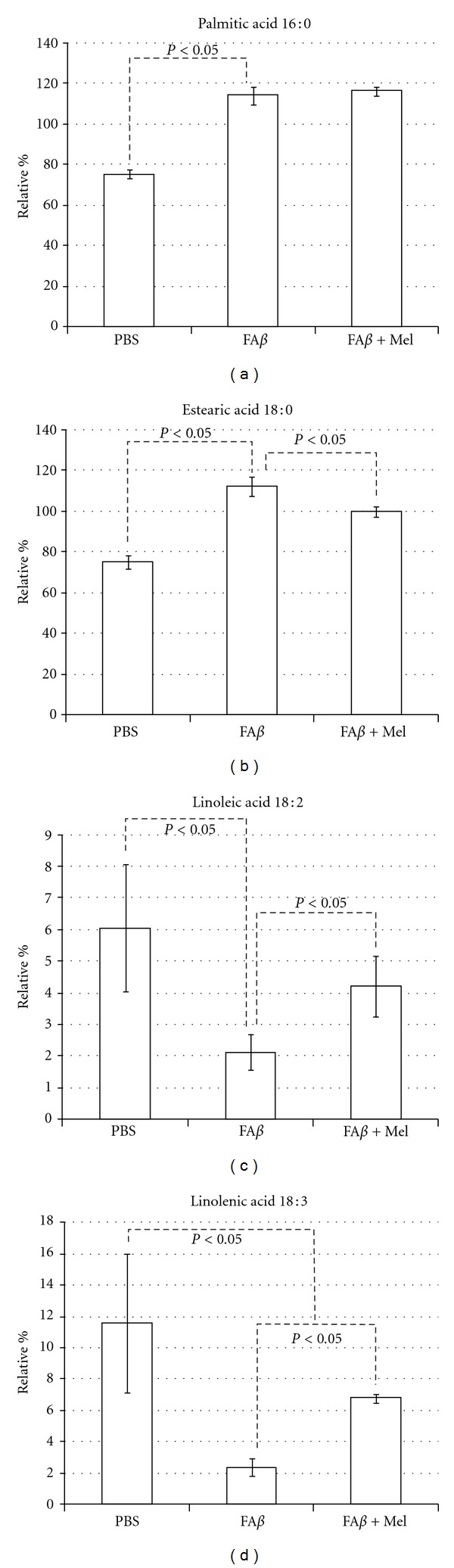
A*β* and H_2_O_2_ (not shown) had similar and highly significant effects on saturated fatty acids particularly on palmitic and estearic acids whose percentages were increased 39 and 37% correspondingly. Linoleic acid was reduced to a third from the control, while linolenic acid was reduced to less than a quart from the control value, as shown. These important effects of A*β* on specific saturated and unsaturated fatty acids affected the unsaturated/saturated (U/S) balance.

A*β* increased palmitic (16 : 0) and estearic (18 : 0) saturated fatty acids, 39 and 37% (*P* < 0.05), correspondingly. Additionally, A*β* reduced linoleic acid (18 : 2) at less than 35% the observed value in the PBS-injected brains (*P* < 0.05) and decreased linolenic acid (18 : 3) value 80% below the observed value in the PBS-injected brains. Additionally, A*β* significantly increased the polyunsaturated arachidonic acid (20 : 4) (*P* < 0.05, from  29.4 ± 2  to 51 ± 2.5). Thus, the elevated increase in saturated plus the severe decrement in linoleic and linolenic acids (Figure 4) was mostly responsible for an alteration in the ratio of unsaturated to saturated fatty acids in membrane phospholipids which is critical to normal cellular function.

**Figure 5:**
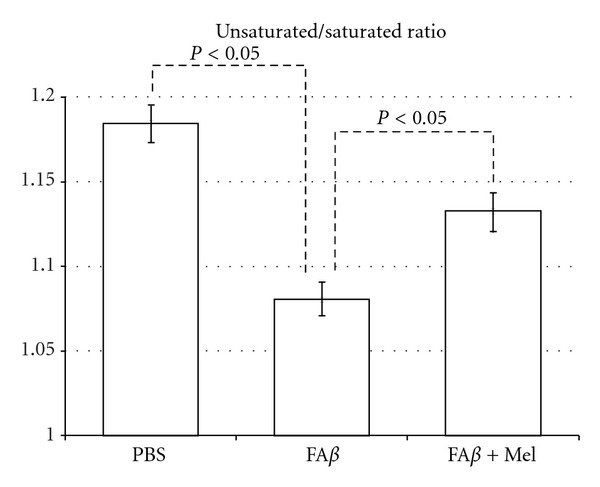
fA*β*-injected brains decreased significantly the U/S ratio, as compared with the PBS-injected brains. However, brains of animals taking oral melatonin showed a U/S ratio closer to the control group.

The lower unsaturated to saturated ratio was observed in fA*β*-injected brains to be even lower than the positive control group of H_2_O_2_, fA*β* and H_2_O_2_ being the groups of study where the overproduction of free radicals was significant (Figure 2). With the use of melatonin, the U/S ratio returned significantly closer to control values (Figure 5), which reflected melatonin's role on each particular fatty acid (Figure 4).

**Figure 6:**
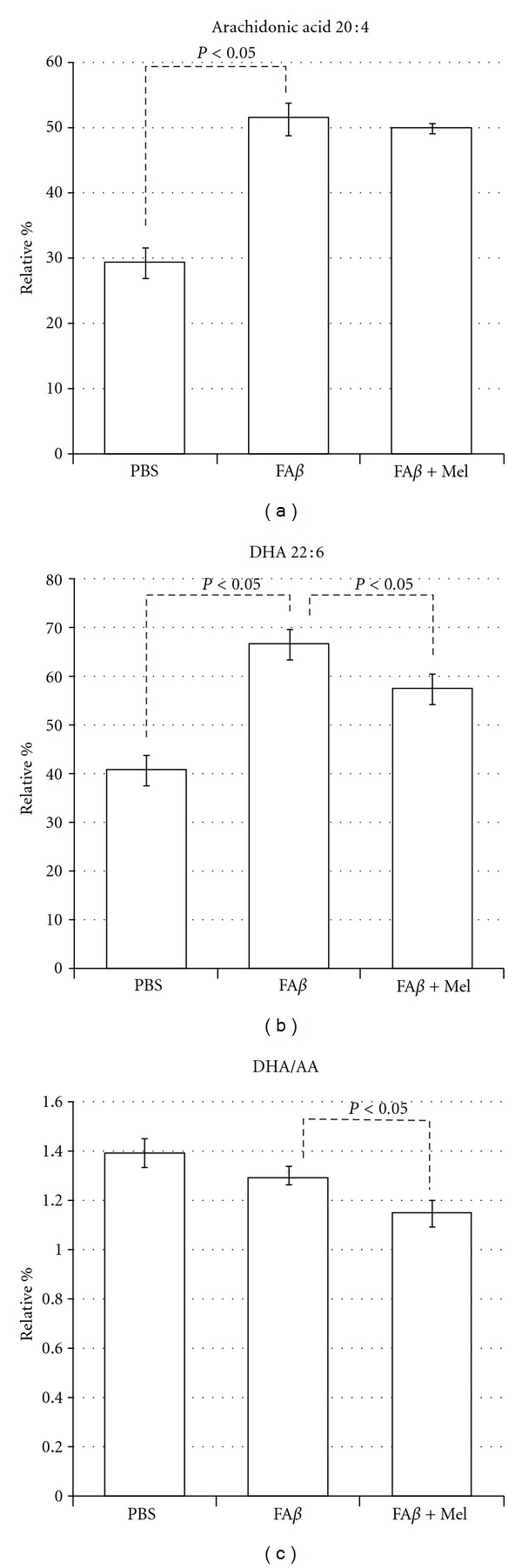
A*β* and H_2_O_2_ (not shown) produced important increases in both n6 and n3 PUFA, which reflects the previous described changes in free fatty acids. A similar increase in DHA and AA allowed the DHA/AA ratio to remain stable, when compared with the PBS group. It is obvious that melatonin reduces the DHA/AA ratio, particularly at the expense of a decrease in DHA levels.

However, the aforementioned showed that a drastic reduction in linolenic acid, a precursor of arachidonic acid (20 : 4, n6), and in linoleic acid, a precursor of docosohexanoic acid (22 : 6, n3), was reflected in the A*β*-induced levels of AA and DHA levels (Figure 6). In the A*β*-injected brains, arachidonic acid rose 20% in relation to the PBS-injected control values (*P* < 0.05), while DHA levels showed approximately 30% increase value (*P* < 0.05). This similar increment in both variables allows that the relationship between these relative values or DHA/AA ratio remains stable in A*β*-injected brains as compared to PBS-injected brains (Figure 6).

**Figure 7:**
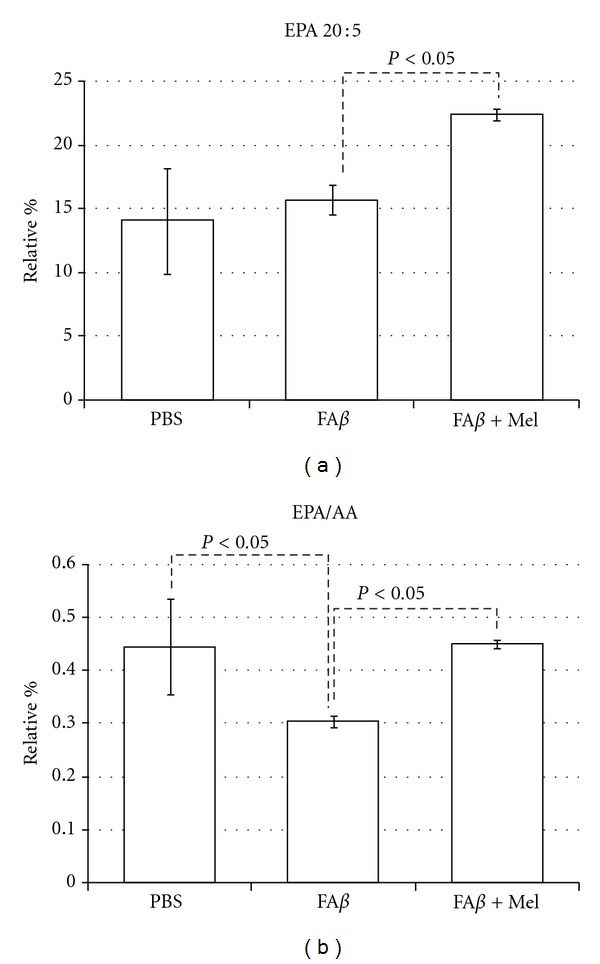
EPA was not to significantly responsive to A*β*. However, in the presence of melatonin and contrary to the results with the other major n3 PUFA, DHA, the relative percentage of EPA rose significantly, which impacted the EPA/AA ratio, as shown.

Except for the palmitic acid and the arachidonic acid, the rest of the A*β*-altered fatty acids tend to return to their basal levels, similar to PBS levels, when the animals were treated with melatonin (fA*β*+Mel group), as shown in Figures 4–6. Another exception was the N3, 20 : 5, polyunsaturated eicosapentaenoic fatty acid (EPA) which seemed to be unaffected by A*β* (Figure 7). However, the EPA-to-AA ratio showed a significant reduction (*P* < 0.05). Interestingly, in A*β*-injected brains from melatonin-treated animals, EPA values were 60% and 43% higher than those observed in PBS- and in A*β*-injected brains. As a result, the EPA to AA ratio was restored (Figure 7).

### 3.5. A*β* Induced Significant Alterations in Mitochondrial Membrane Phospholipids

**Figure 8:**
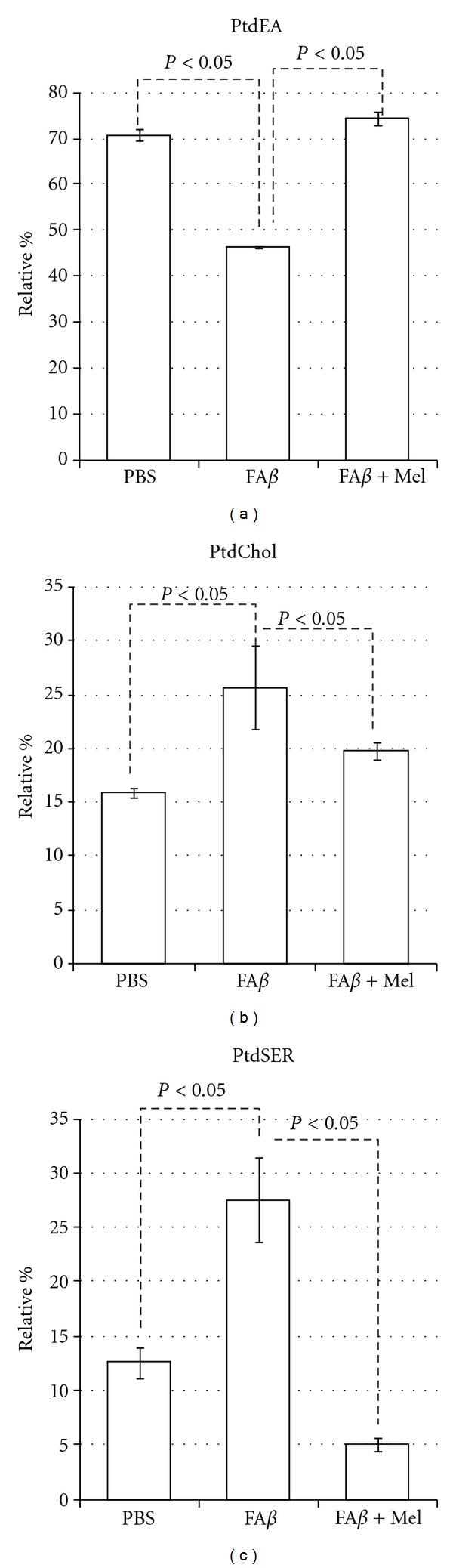
A*β* decreased significantly the PtdEA levels and increased the levels of PtdCHOL and PtdSER, the latter with a 5-fold increment. Results are expressed in relative percentage ± standard error.

Thus, while the effect of phosphatidyl ethanolamine (PtdEA) levels was reduced by a third in the fA*β* group (*P* < 0.05), levels of phosphatidyl choline (PtdCHOL) were increased 40% (*P* < 0.05), but the phosphatidyl serine (PtdSER) values reached 120% as compared to PBS-injected brains (Figure 8). On the contrary, brains of animals treated with melatonin showed PtdEA levels similar to PBS-injected brains, while levels of PtdSER were significantly reduced with melatonin even beyond the control values (*P* < 0.05). PtdCHOL levels were also significantly reduced in presence of melatonin (Figure 8).

### 3.6. Variations in Cholesterol Content Follow the Same Pattern As Variations in Free Radicals

**Figure 9:**
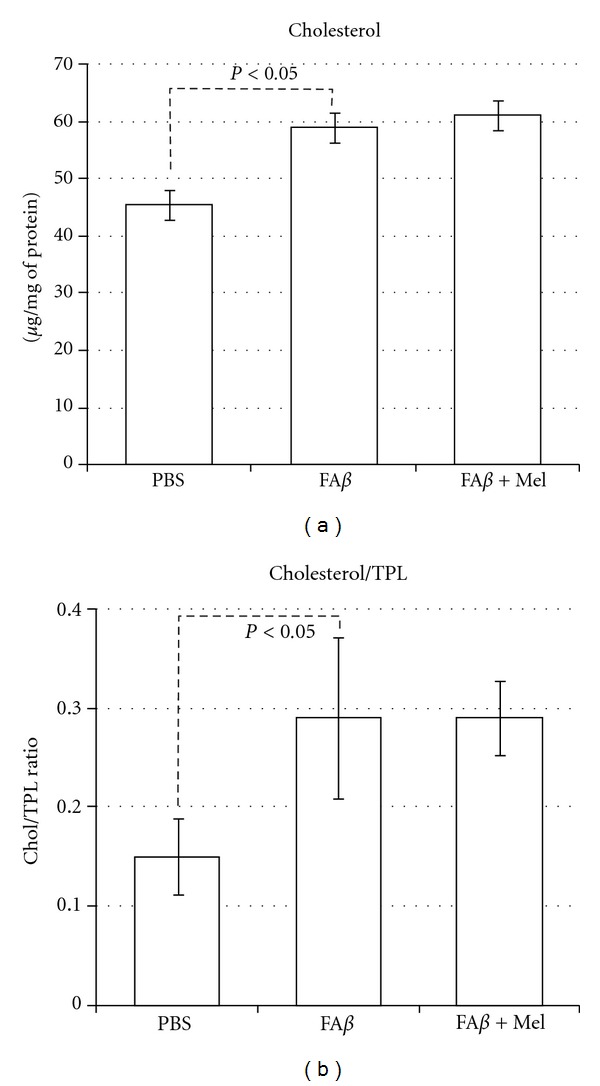
Cholesterol content in mitochondrial membranes is significantly increased in fA*β* injected brains. The H_2_O_2_ control group (data not shown) and the fA*β* experimental group, which showed the more important overproduction of free radicals and the lowest membrane fluidity, coincide with the highest cholesterol content. However, in spite of its ability to scavenge free radicals and restore membrane fluidity, melatonin was unable to reduce cholesterol content in mitochondrial membrane. Compared according to their relative values, cholesterol and total phospholipids ratio was significantly altered.

The lowest cholesterol values were found in the PBS-injected brains and the highest values were observed in the H_2_O_2_-injected brains (*P* < 0.05). The concentration of cholesterol in fA*β*-injected brains was significantly higher (59.6 ± 5.7 versus 47.6 ± 5.2 *μ*g/mg of protein, *P* < 0.05) than that in the control brains treated with PBS; this value was only 66% the value in H_2_O_2_-injected brains (59.6 ± 5.7 versus 90.24 ± 11, data not shown). In spite of the significant increase in the membrane fluidity observed in fA*β*+Mel brains, this change was apparently not related to the cholesterol content since melatonin did not change the levels of cholesterol in fA*β*-injected brains (fA*β*  60 ± 6  versus fA*β*+Mel 61 ± 7) (Figure 9). The cholesterol-to-phospholipid ratio, on the other side, which reflects a loss of phospholipid and membrane rigidity, was significantly altered by A*β*, with the inability of melatonin to return it to the level found in PBS-injected brains (Figure 9). 

## 4. Discussion

 Lipid and fatty acid changes have been studied in plasma membranes, especially in both postmortem AD brain and transgenic mice. These changes have been associated with aging, A*β* deposits, dementia, and even mild cognitive impairment. Other experiments have been carried out* in vitro* by using purified synaptosomal plasma or mitochondrial membranes. To our knowledge, no studies have focused on mitochondrial membranes in response to *in vivo* extracellular deposits of fibrillar A*β*. In spite of its known ability to induce oxidative stress, alterations in lipid content of mitochondrial membranes induced by extracellular A*β* differed from those induced by H_2_O_2_.

 Mitochondrial dysfunction has been related to oxidative stress in neurodegeneration as both cause and effect. At the same time, there is increasing evidence for membrane lipid, fatty acid, and cholesterol interactions with A*β*. These interactions have significant consequences in the pathogenesis of Alzheimer's disease. Our original aim was to demonstrate that extracellular deposits of A*β*, *in vivo,* would be able to induce mitochondrial failure as well as changes in mitochondrial lipid composition as a consequence of its ability to induce oxidative stress. H_2_O_2_ was chosen as positive control since endogenous hydrogen peroxide has been indicated as a secondary messenger mediating the intracellular effects of A*β* extracellular deposits. Furthermore, a relationship between the accumulation of A*β* monomers and oligomers and H_2_O_2_ production in the mitochondria of Tg2576 mice has been reported. However, we found that important A*β*-induced alterations were significantly different from those induced by H_2_O_2_. AA levels, for example, were significantly higher in the fA*β* group than those in the H_2_O_2_ group (*P* < 0.05). The ratio U/S was not affected by H_2_O_2_, basically due to minimal effects on or to compensations between decrements and increments in one or another group of saturated versus unsaturated fatty acids. A*β*, increasing saturated fatty acids and decreasing unsaturated fatty acids, caused a persistent and significant decrement of the U/S ratio (Figure 5). This finding agrees with another work where the ability of A*β* to interfere with the Δ-9 desaturase enzyme was observed. Δ-9 desaturase introduces the first double bond between carbon 9 and 10 of palmitoyl (16 : 0) or stearyl (18 : 0) Co A to form palmitoleic (16 : 1) or oleic (18 : 1) acids [19].

 This profound fatty acid imbalance was reflected in phospholipid levels, in such a manner that H_2_O_2_ did not affect the levels of PtdEA, but A*β* reduced significantly this phospholipid. However, the most severe effect of A*β* was observed in PtdSER, causing a 5-fold increment, whereas H_2_O_2_ did not affect this parameter.

**Figure 10:**
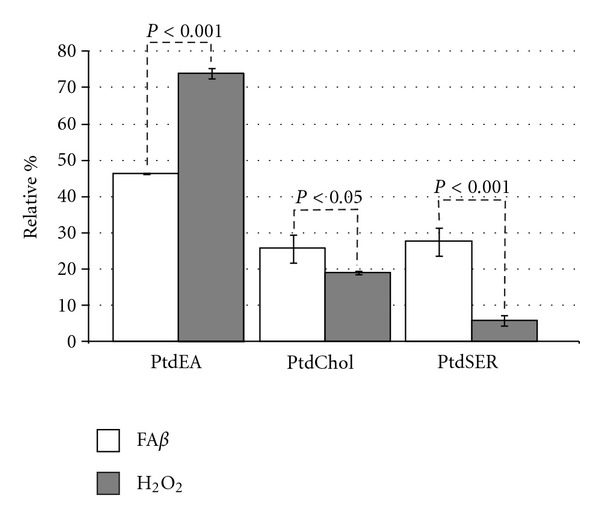
Significant differences between A*β*-injected brains and H_2_O_2_-injected brains.

 There seems to be a different mechanism of damage. While the strict oxidative damage, represented by our positive control group H_2_O_2_, caused the highest increment in PtdEA levels, A*β* reduced PtdEA levels and, on the contrary, increased very significantly the PtdSER levels and, less important but also significantly, the PtdCHOL levels (Figure 10).

 Thus, A*β* deleterious effects were not oxidative stress related—or at least not completely explained by oxidative alterations—which is evident when comparing the differing effects of H_2_O_2_ and A*β* on membrane lipids (*P* < 0.05). Fatty acid (FA) composition of phospholipids determines biophysical (and functional) characteristics of membranes (e.g., membrane fluidity) and plays an important role in cellular integrity and intra- and intercellular communication. We found a significant (*P* < 0.001) inverse correlation (*r* ^2^ = −0.74) between mitochondrial membrane fluidity and cholesterol content. Indeed, we found that A*β* and H_2_O_2_ caused the more severe oxidative stress, the lowest membrane fluidity, and the highest cholesterol content. However, in the A*β* group the reduction of oxidative stress seemed not to affect the cholesterol increment, although A*β* had a more severe effect on fatty acids (Figures 5, 6, and 7) and phospholipid redistribution (Figure 10).

 A tendency of cholesterol to aggregate into clusters at a cholesterol/phospholipid ratio of greater than 0.3 is known since 1972 [50], and we have found a 0.3 ± .08 cholesterol to phospholipid ratio in fA*β*-injected brains due principally to a severe decrement in PtdEA against an increment in cholesterol content (Figures 8 and 9). Cholesterol aggregates in membranes are a well-known characteristic of A*β*-induced damage [30, 51, 52]. High-cholesterol diet has been associated with increased deposits of A*β* [53], and we have found [54] that animals fed with a cholesterol-enriched diet presented a significant increase in mitochondrial structural damage linked to severe dysfunction of this organelle. It was noticeable that, according to our results, melatonin could not impair this cholesterol re-arrangement but was able to induce a significant increment in membrane fluidity (Figure 3). This phenomenon illustrates the role of lipid peroxidation and the reaccommodation of phospolipids, particularly PtdSer and PtdEA, along the membranes as determinants of membrane fluidity, beyond the role of cholesterol [55].

 A*β*_42_ oligomers accumulate more slowly and in reduced amount at the plasma membranes of fibroblasts from familial AD (FAD) patients enriched in cholesterol [56]. On the other side, it is also reported that A*β* binds lipids, but with a higher affinity for cholesterol than PtdCHOL or saturated fatty acids [51]. We may therefore speculate, according to our results, that the cholesterol rearrangement observed in brain cells may be a defensive response against oxidative stress, with a secondary effect, A*β* binding.

 Specific alterations in fatty acids have been related to A*β* pathogenesis. For example, unsaturated fatty acids oleic acid, and linoleic acid, have been shown to increase the *γ*-secretase activity and A*β* levels, as evaluated in PSwt-1 cells, which contains the wild-type human presenilin 1 (PS1) and wild-type human APP full-length cDNAs, [11]. According to our results, A*β* decreased severely the content of linoleic acid (6.5 mol% in PBS-injected brains versus 2.12 mol% in A*β*-injected brains, *P* < 0.05), as observed in old Wistar rat brain, which implies that this effect occurs regardless of the ApoE phenotype.

 The importance of the ApoE phenotype involves the carrying of proteins in combination with lipids to form lipoprotein particles with hydrophobic lipids at the core and hydrophilic side chains made of amino acids. ApoE also aids the transport of triglyceride, phospholipid, and cholesterol into cells, by mediating the binding, internalization, and catabolism of lipoprotein particles [57]. ApoE is considered a risk factor in AD because 40–65% of AD patients have at least one copy of the 4 alleles; although the exact mechanism of this feature remains to be fully determined, an interaction with amyloid insoluble protein aggregates or with APP seems to be involved [58, 59]. How ApoE controls brain lipids and how this regulation may impact the clearance of A*β* or the progression of damage are less clear [60]. In postmortem brain samples, no significant difference in lipids or fatty acids was found between AD patients classified as homozygous for ApoE4 and those classified as heterozygous or having no ApoE4 [10, 61]. Thus, ApoE genotype on fatty acids and lipid composition and/or its distribution in brain cell membranes seem to have no significance and would not bias our results. Additionally, by comparing the association of human, rat, and rabbit ApoE with A*β*, a similar lack of affinity for A*β* between rat ApoE and human ApoE4 has been reported [62]. Thus, rat ApoE, the same as the AD-related ApoE4, does not form complex with A*β*.

 Another fatty acid whose relationship with A*β* pathogeny has been widely studied is AA. This N6 PUFA is an agonist of proinflammatory pathways, which additionally as has been reported increase the levels of A*β* [63]. It is known also that A*β* oligomers trigger neuronal apoptosis by early activation of a cPLA2-dependent pathway leading to production of AA [14]. We found that the AA precursor linoleic acid was reduced while AA was increased by A*β*, which supports the proposal that A*β* paves its own way by changing the quality and distribution of lipid membranes.

 Herein we report important alterations in mitochondrial membranes following the intracerebral injection of A*β*. There is important in this context the relationship between n6 and n3 PUFA, particularly the relationship between the proinflammatory AA with its counterparts DHA and EPA. EPA and DHA differ in their effects on plasma lipid profiles, gene expression, and neural membrane structure. EPA downregulates the enzymes involved in DHA synthesis and decreases DHA synthesis from its precursor, *α*-linolenic acid [64]. We have found that A*β* increased both DHA and AA, while the levels of EPA remained stable, but the treatment with melatonin, which did not affect the levels of AA, was able to increase very importantly EPA. EPA has been reported as anti-inflammatory upon several conditions and different cell types, but importantly in aging and A*β*-induced neuroinflammation [65–67]. Specifically EPA is linked to a modulatory role in microglial activity [68]. We have reported a remarkable reduction in microglial activity in rats intracerebrally injected with A*β* but under melatonin treatment [4]. 

 Even though being examined in a different context, there is a report where melatonin was found protective of AA, DHA, and EPA. Arachidonic acid was protected more efficiently than DHA and EPA at all the melatonin concentrations examined when rat liver microsomes were incubated with ascorbic acid [69], which is quite similar to our results (Figures 6 and 7). This phenomenon is linked to protection against lipid peroxidation, a remarkable ability of melatonin supported by its amphoteric nature as well as its ability to cross the blood-brain barrier (BBB) and enter into the central nervous system [70].

 Evaluated in cortical synaptosomes from gerbils, a loss of phospholipid asymmetry induced by A*β*_1–42_ has been reported [71]. This phenomenon implies the oxidative modification of the flippase enzyme by reactive alkenals which causes externalization of PtdSER and the subsequent phospholipid asymmetry, which in turn causes membrane dysfunction, Ca^++^ massive influx, and apoptosis. The anionic PtdSER also may increase the fibrillization of A*β* [72]. We found that A*β* causes a 120% increase in PtdSER levels. However PtdSER levels in brains of animals which received melatonin treatment decreased 5 times compared to brains from animals without melatonin treatment.

## 5. Conclusions

 The relationship between membrane lipids with A*β* is usually focused on how lipids may allow, facilitate, or even induce the amyloidogenic processing of APP. This relationship is also explored to explain how A*β* causes cellular dysfunction.

 Our approach to the *in vivo *study of the A*β*_1–42_ peptide, the predominantly neurotoxic form of A*β*, was to inject the peptide directly into the hippocampus and then examine the relationship with membrane lipids, in order to explain how A*β* may penetrate the cell and then approach to mitochondria and cause the well-known severe dysfunction of this organelle.

 The intracellular amyloid cascade is, of course, widely studied and elegantly explained [73–75]. It is also likely that the pathogenically critical process of A*β* oligomerization may begin intraneuronally and the energy hypometabolism may appear before the presence of senile plaques or neurofibrillary tangles [76]. However, without discarding the previous statements, there is evidence to consider the extracellular A*β* as the principal source of intracellular A*β*, given the huge amounts of A*β* in aggregates, the physical properties of this peptide, and its ability to alter fatty acids and lipids on membranes, either because of its pro-oxidant activity or because of its physical interactions with lipids.

 We reported how exogenous A*β* forms deposits in the extracellular space, then presents inside the cells—particularly through the axons causing demyelination, which agrees with other reports [5, 6]—and, finally, how A*β* is found inside mitochondria where it causes severe structural damage linked to free radical overproduction and significant alterations in mitochondrial membrane lipids.

By using melatonin, it is possible to ameliorate the membrane fluidity without affecting cholesterol content in membranes, while it restores the balance of lipids. Importantly, melatonin reduces the negatively charged PtdSER in membranes and, by this means, might impair the toxicity of A*β*. Another important feature is how melatonin may increase EPA content in membranes, restoring the EPA/AA ratio, a phenomenon widely known by its anti-inflammatory effects. Melatonin restores membrane structure and functionality, an effect which exclusively could not be attributed to its antioxidant capacity.

## References

[1] De Pietri Tonelli D, Mihailovich M, Di Cesare A, Codazzi F, Grohovaz F, Zacchetti D (2004). Translational regulation of BACE-1 expression in neuronal and non-neuronal cells. *Nucleic Acids Research*.

[2] Maggio JE, Mantyh PW (1996). Brain amyloid—a physicochemical perspective. *Brain Pathology*.

[3] Yatin SM, Varadarajan S, Link CD, Butterfield DA (1999). In vitro and in vivo oxidative stress associated with Alzheimer’s amyloid *β*-peptide (1–42). *Neurobiology of Aging*.

[4] Rosales-Corral S, Tan DX, Reiter RJ (2003). Orally administered melatonin reduces oxidative stress and proinflammatory cytokines induced by amyloid-*β* peptide in rat brain: a comparative, in vivo study versus vitamin C and E. *Journal of Pineal Research*.

[5] Lassmann H (2011). Mechanisms of neurodegeneration shared between multiple sclerosis and Alzheimer’s disease. *Journal of Neural Transmission*.

[6] Mitew S, Kirkcaldie MTK, Halliday GM, Shepherd CE, Vickers JC, Dickson TC (2010). Focal demyelination in Alzheimer’s disease and transgenic mouse models. *Acta Neuropathologica*.

[7] Brown MS, Ye J, Rawson RB, Goldstein JL (2000). Regulated intramembrane proteolysis: a control mechanism conserved from bacteria to humans. *Cell*.

[8] Patil S, Sheng L, Masserang A, Chan C (2006). Palmitic acid-treated astrocytes induce BACE1 upregulation and accumulation of C-terminal fragment of APP in primary cortical neurons. *Neuroscience Letters*.

[9] Patil S, Chan C (2005). Palmitic and stearic fatty acids induce Alzheimer-like hyperphosphorylation of tau in primary rat cortical neurons. *Neuroscience Letters*.

[10] Igarashi M, Ma K, Gao F, Kim HW, Rapoport SI, Rao JS (2011). Disturbed choline plasmalogen and phospholipid fatty acid concentrations in Alzheimer’s disease prefrontal cortex. *Journal of Alzheimer’s Disease*.

[11] Liu Y, Yang L, Conde-Knape K, Beher D, Shearman MS, Shachter NS (2004). Fatty acids increase presenilin-1 levels and *γ*-secretase activity in PSwt-1 cells. *Journal of Lipid Research*.

[12] Lovell MA, Xie C, Markesbery WR (1998). Decreased glutathione transferase activity in brain and ventricular fluid in Alzheimer’s disease. *Neurology*.

[13] Esterbauer H, Schaur RJ, Zollner H (1991). Chemistry and Biochemistry of 4-hydroxynonenal, malonaldehyde and related aldehydes. *Free Radical Biology and Medicine*.

[14] Kriem B, Sponne I, Fifre A (2005). Cytosolic phospholipase A2 mediates neuronal apoptosis induced by soluble oligomers of the amyloid-*β* peptide. *The FASEB Journal*.

[15] Lim GP, Calon F, Morihara T (2005). A diet enriched with the omega-3 fatty acid docosahexaenoic acid reduces amyloid burden in an aged Alzheimer mouse model. *Journal of Neuroscience*.

[16] Sahlin C, Pettersson FE, Nilsson LNG, Lannfelt L, Johansson AS (2007). Docosahexaenoic acid stimulates non-amyloidogenic APP processing resulting in reduced A*β* levels in cellular models of Alzheimer’s disease. *European Journal of Neuroscience*.

[17] Umezawa M, Kogishi K, Tojo H (1999). High-linoleate and high-*α*-linolenate diets affect learning ability and natural behavior in SAMR1 mice. *Journal of Nutrition*.

[18] Bazan NG (2005). Synaptic signaling by lipids in the life and death of neurons. *Molecular Neurobiology*.

[19] Morley JE, Farr SA, Kumar VB, Banks WA (2002). Alzheimer’s disease through the eye of a mouse: acceptance lecture for the 2001 Gayle A. Olson and Richard D. Olson prize. *Peptides*.

[20] McLaurin J, Chakrabartty A (1997). Characterization of the interactions of Alzheimer *β*-amyloid peptides with phospholipid membranes. *European Journal of Biochemistry*.

[21] Terzi E, Holzemann G, Seelig J (1995). Self-association of *β*-amyloid peptide (1–40) in solution and binding to lipid membranes. *Journal of Molecular Biology*.

[22] Simakova O, Arispe NJ (2007). The cell-selective neurotoxicity of the Alzheimer’s A*β* peptide is determined by surface phosphatidylserine and cytosolic ATP levels. Membrane binding is required for A*β* toxicity. *Journal of Neuroscience*.

[23] Hampton MB, Vanags DM, Pörn-Ares MI, Orrenius S (1996). Involvement of extracellular calcium in phosphatidylserine exposure during apoptosis. *FEBS Letters*.

[24] Rosales-Corral SA, Acuna-Castroviejo D, Coto-Montes A (2012). Alzheimer's disease: pathological mechanisms and the beneficial role of melatonin. *Journal of Pineal Research*.

[25] Lee G, Pollard HB, Arispe N (2002). Annexin 5 and apolipoprotein E2 protect against Alzheimer’s amyloid-*β*-peptide cytotoxicity by competitive inhibition at a common phosphatidylserine interaction site. *Peptides*.

[26] Lai Z, Colón W, Kelly JW (1996). The acid-mediated denaturation pathway of transthyretin yields a conformational intermediate that can self-assemble into amyloid. *Biochemistry*.

[27] de Planque MRR, Raussens V, Contera SA (2007). Beta-Sheet structured beta-amyloid(1–40) perturbs phosphatidylcholine model membranes. *Journal of Molecular Biology*.

[28] Eckert GP, Kirsch C, Leutz S, Wood WG, Müller WE (2003). Cholesterol modulates amyloid beta-peptide's membrane interactions. *Pharmacopsychiatry*.

[29] Gibson Wood W, Eckert GP, Igbavboa U, Muller WE (2003). Amyloid beta-protein interactions with membranes and cholesterol: causes or casualties of Alzheimer's disease. *Biochim Biophys Acta*.

[30] Kakio A, Nishimoto SI, Yanagisawa K, Kozutsumi Y, Matsuzaki K (2001). Cholesterol-dependent formation of GM1 ganglioside-bound amyloid beta-protein, an endogenous seed for Alzheimer amyloid. *The Journal of Biological Chemistry*.

[31] Subasinghe S, Unabia S, Barrow CJ, Mok SS, Aguilar MI, Small DH (2003). Cholesterol is necessary both for the toxic effect of A*β* peptides on vascular smooth muscle cells and for A*β* binding to vascular smooth muscle cell membranes. *Journal of Neurochemistry*.

[32] Buchsteiner A, Hauss T, Dante S, Dencher NA (2010). Alzheimer's disease amyloid-beta peptide analogue alters the ps-dynamics of phospholipid membranes. *Biochim Biophys Acta*.

[33] Qiu L, Buie C, Reay A, Vaughn MW, Cheng KH (2011). Molecular dynamics simulations reveal the protective role of cholesterol in beta-amyloid protein-induced membrane disruptions in neuronal membrane mimics. *Journal of Physical Chemistry B*.

[34] D'Errico G, Vitiello G, Ortona O, Tedeschi A, Ramunno A, D'Ursi AM (2008). Interaction between Alzheimer's Abeta(25-35) peptide and phospholipid bilayers: the role of cholesterol. *Biochim Biophys Acta*.

[35] Mecocci P, Cherubini A, Beal MF (1996). Altered mitochondrial membrane fluidity in AD brain. *Neuroscience Letters*.

[36] Chen JJ, Yu BP (1994). Alterations in mitochondrial membrane fluidity by lipid peroxidation products. *Free Radical Biology and Medicine*.

[37] Leaden P, Barrionuevo J, Catalá A (2002). The protection of long chain polyunsaturated fatty acids by melatonin during nonenzymatic lipid peroxidation of rat liver microsomes. *Journal of Pineal Research*.

[38] Feng Z, Qin C, Chang Y, Zhang JT (2006). Early melatonin supplementation alleviates oxidative stress in a transgenic mouse model of Alzheimer’s disease. *Free Radical Biology and Medicine*.

[39] Gavazza M, Catalá A (2003). Melatonin preserves arachidonic and docosapentaenoic acids during ascorbate-Fe^2+^ peroxidation of rat testis microsomes and mitochondria. *International Journal of Biochemistry and Cell Biology*.

[40] Hensley K, Carney JM, Mattson MP (1994). A model for *β*-amyloid aggregation and neurotoxicity based on free radical generation by the peptide: relevance to Alzheimer disease. *Proceedings of the National Academy of Sciences of the United States of America*.

[41] Liang JJ, Gu CL, Kacher ML, Foote CS (1983). Chemistry of singlet oxygen. 45. Mechanism of the photooxidation of sulfides. *Journal of the American Chemical Society*.

[42] Milton NGN (2004). Role of hydrogen peroxide in the aetiology of Alzheimer's disease: implications for treatment. *Drugs and Aging*.

[43] Behl C, Davis JB, Lesley R, Schubert D (1994). Hydrogen peroxide mediates amyloid *β* protein toxicity. *Cell*.

[44] Shi C, Wu F, Xu J (2010). H2O2 and PAF mediate A*β*1–42-induced Ca^2+^ dyshomeostasis that is blocked by EGb761. *Neurochemistry International*.

[45] Manczak M, Anekonda TS, Henson E, Park BS, Quinn J, Reddy PH (2006). Mitochondria are a direct site of A*β* accumulation in Alzheimer’s disease neurons: implications for free radical generation and oxidative damage in disease progression. *Human Molecular Genetics*.

[46] Weldon DT, Rogers SD, Ghilardi JR (1998). Fibrillar *β*-amyloid induces microglial phagocytosis, expression of inducible nitric oxide synthase, and loss of a select population of neurons in the rat CNS *in vivo*. *Journal of Neuroscience*.

[47] Ishii K, Muelhauser F, Liebl U (2000). Subacute NO generation induced by Alzheimer’s *β*-amyloid in the living brain: reversal by inhibition of the inducible NO synthase. *The FASEB Journal*.

[48] Paxinos G (1984). *The Rat Nervous System*.

[49] Lowry OH, Rosebrough NJ, Farr AL, Randall RJ (1951). Protein measurement with the Folin phenol reagent. *The Journal of Biological Chemistry*.

[50] Engelman DM, Rothman JE (1972). The planar organization of lecithin-cholesterol bilayers. *The Journal of Biological Chemistry*.

[51] Avdulov NA, Chochina SV, Igbavboa U, Warden CS, Vassiliev AV, Wood WG (1997). Lipid binding to amyloid *β*-peptide aggregates: preferential binding of cholesterol as compared with phosphatidylcholine and fatty acids. *Journal of Neurochemistry*.

[52] Kakio A, Nishimoto S, Kozutsumi Y, Matsuzaki K (2003). Formation of a membrane-active form of amyloid beta-protein in raft-like model membranes. *Biochemical and Biophysical Research Communications*.

[53] Ginsberg L, Xuereb JH, Gershfeld NL (1998). Membrane instability, plasmalogen content, and Alzheimer’s disease. *Journal of Neurochemistry*.

[54] Rosales-Corral SA, Acuna-Castroviejo D, Tan D-X (2012). Alzheimer's disease: pathological mechanisms and the beneficial role of melatonin. *Journal Pineal Research*.

[55] Shinitzky M, Barenholz Y (1978). Fluidity parameters of lipid regions determined by fluorescence polarization. *Biochimica et Biophysica Acta*.

[56] Pensalfini A, Zampagni M, Liguri G (2011). Membrane cholesterol enrichment prevents A*β*-induced oxidative stress in Alzheimer’s fibroblasts. *Neurobiology of Aging*.

[57] Eichner JE, Dunn ST, Perveen G, Thompson DM, Stewart KE, Stroehla BC (2002). Apolipoprotein E polymorphism and cardiovascular disease: a HuGE review. *American Journal of Epidemiology*.

[58] Sadowski MJ, Pankiewicz J, Scholtzova H (2006). Blocking the apolipoprotein E/amyloid-*β* interaction as a potential therapeutic approach for Alzheimer’s disease. *Proceedings of the National Academy of Sciences of the United States of America*.

[59] Haß S, Fresser F, Köchl S, Beyreuther K, Utermann G, Baier G (1998). Physical interaction of ApoE with amyloid precursor protein independent of the amyloid A*β* region in vitro. *The Journal of Biological Chemistry*.

[60] Arold S, Sullivan P, Bilousova T (2012). Apolipoprotein E level and cholesterol are associated with reduced synaptic amyloid beta in Alzheimer's disease and apoE TR mouse cortex. *Acta Neuropathologica*.

[61] Fraser T, Tayler H, Love S (2010). Fatty acid composition of frontal, temporal and parietal neocortex in the normal human brain and in Alzheimer’s disease. *Neurochemical Research*.

[62] LaDu MJ, Lukens JR, Reardon CA, Getz GS (1997). Association of human, rat, and rabbit apolipoprotein E with beta-amyloid. *Journal of Neuroscience Research*.

[63] Amtul Z, Uhrig M, Beyreuther K (2011). Additive effects of fatty acid mixtures on the levels and ratio of amyloid *β*40/42 peptides differ from the effects of individual fatty acids. *Journal of Neuroscience Research*.

[64] Langelier B, Alessandri JM, Perruchot MH, Guesnet P, Lavialle M (2005). Changes of the transcriptional and fatty acid profiles in response to n-3 fatty acids in SH-SY5Y neuroblastoma cells. *Lipids*.

[65] Babcock T, Helton WS, Espat NJ (2000). Eicosapentaenoic acid (EPA): an antiinflammatory *ω*-3 fat with potential clinical applications. *Nutrition*.

[66] Lynch AM, Loane DJ, Minogue AM (2007). Eicosapentaenoic acid confers neuroprotection in the amyloid-*β* challenged aged hippocampus. *Neurobiology of Aging*.

[67] Minogue AM, Lynch AM, Loane DJ, Herron CE, Lynch MA (2007). Modulation of amyloid-*β*-induced and age-associated changes in rat hippocampus by eicosapentaenoic acid. *Journal of Neurochemistry*.

[68] Bernardo A, Levi G, Minghetti L (2000). Role of the peroxisome proliferator-activated receptor-*γ* (PPAR-*γ*) and its natural ligand 15-deoxy-Δ(12,14)-prostaglandin J2 in the regulation of microglial functions. *European Journal of Neuroscience*.

[69] Tang PL, Xu MF, Qian ZM (1997). Differential behaviour of cell membranes towards iron-induced oxidative damage and the effects of melatonin. *Biological Signals*.

[70] Reiter RJ, Cabrera J, Sainz RM, Mayo JC, Manchester LC, Tan DX (1999). Melatonin as a pharmacological agent against neuronal loss in experimental models of Huntington’s disease, Alzheimer’s disease and Parkinsonism. *Annals of the New York Academy of Sciences*.

[71] Abdul HM, Butterfield DA (2005). Protection against amyloid beta-peptide (1–42)-induced loss of phospholipid asymmetry in synaptosomal membranes by tricyclodecan-9-xanthogenate (D609) and ferulic acid ethyl ester: implications for Alzheimer’s disease. *Biochimica et Biophysica Acta*.

[72] Chauhan A, Ray I, Chauhan VPS (2000). Interaction of amyloid beta-protein with anionic phospholipids: possible involvement of Lys28 and C-terminus aliphatic amino acids. *Neurochemical Research*.

[73] Yang AJ, Chandswangbhuvana D, Shu T, Henschen A, Glabe CG (1999). Intracellular accumulation of insoluble, newly synthesized A*β*n-42 in amyloid precursor protein-transfected cells that have been treated with A*β*1-42. *The Journal of Biological Chemistry*.

[74] Hansson Petersen CA, Alikhani N, Behbahani H (2008). The amyloid *β*-peptide is imported into mitochondria via the TOM import machinery and localized to mitochondrial cristae. *Proceedings of the National Academy of Sciences of the United States of America*.

[75] Swerdlow RH, Khan SM (2009). The Alzheimer’s disease mitochondrial cascade hypothesis: an update. *Experimental Neurology*.

[76] Atamna H, Frey WH (2007). Mechanisms of mitochondrial dysfunction and energy deficiency in Alzheimer’s disease. *Mitochondrion*.

